# A new look at the LTR retrotransposon content of the chicken genome

**DOI:** 10.1186/s12864-016-3043-1

**Published:** 2016-08-30

**Authors:** Andrew S. Mason, Janet E. Fulton, Paul M. Hocking, David W. Burt

**Affiliations:** 1 The Roslin Institute and Royal (Dick) School of Veterinary Studies, The University of Edinburgh, Easter Bush, Midlothian, EH25 9RG UK; 2 Hy-Line International, 1915 Sugar Grove Avenue, Dallas Grove, IA 50063 USA

**Keywords:** LTR retrotransposon, Endogenous retrovirus, ERV, Chicken, LocaTR identification pipeline

## Abstract

**Background:**

LTR retrotransposons contribute approximately 10 % of the mammalian genome, but it has been previously reported that there is a deficit of these elements in the chicken relative to both mammals and other birds. A novel LTR retrotransposon classification pipeline, LocaTR, was developed and subsequently utilised to re-examine the chicken LTR retrotransposon annotation, and determine if the proposed chicken deficit is biologically accurate or simply a technical artefact.

**Results:**

Using LocaTR 3.01 % of the chicken galGal4 genome assembly was annotated as LTR retrotransposon-derived elements (nearly double the previous annotation), including 1,073 that were structurally intact. Element distribution is significantly correlated with chromosome size and is non-random within each chromosome. Elements are significantly depleted within coding regions and enriched in gene sparse areas of the genome. Over 40 % of intact elements are found in clusters, unrelated by age or genera, generally in poorly recombining regions. The transcription of most LTR retrotransposons were suppressed or incomplete, but individual domain and full length retroviral transcripts were produced in some cases, although mostly with regularly interspersed stop codons in all reading frames. Furthermore, RNAseq data from 23 diverse tissues enabled greater characterisation of the co-opted endogenous retrovirus *Ovex1*. This gene was shown to be expressed ubiquitously but at variable levels across different tissues. LTR retrotransposon content was found to be very variable across the avian lineage and did not correlate with either genome size or phylogenetic position. However, the extent of previous, species-specific LTR retrotransposon annotation appears to be a confounding factor.

**Conclusions:**

Use of the novel LocaTR pipeline has nearly doubled the annotated LTR retrotransposon content of the chicken genome compared to previous estimates. Further analysis has described element distribution, clustering patterns and degree of expression in a variety of adult tissues, as well as in three embryonic stages. This study also enabled better characterisation of the co-opted gamma retroviral *envelope* gene *Ovex1*. Additionally, this work suggests that there is no deficit of LTR retrotransposons within the Galliformes relative to other birds, or to mammalian genomes when scaled for the three-fold difference in genome size.

**Electronic supplementary material:**

The online version of this article (doi:10.1186/s12864-016-3043-1) contains supplementary material, which is available to authorized users.

## Background

Long Terminal Repeat (LTR) retrotransposons are a diverse group of autonomous, Eukaryotic repeat elements which share a largely conserved, virus-like gene structure flanked by the eponymous LTRs. These elements are primarily intracellular and propagate by retrotransposition in a ‘copy-and-paste’ manner, often resulting in high genomic copy number. In addition, multiple, independent acquisitions of the *env* gene have enabled some of this group, such as the retroviruses, to become extracellular whilst still reliant on chromosomal reintegration for their replication. When such integrations occur in the germline they are inherited vertically in a Mendelian fashion and are said to have become endogenous retroviruses (ERVs). In other LTR retrotransposon groups *env* gene acquisition has been much rarer (Copia/Ty1, Gypsy/Ty3 and Bel/Pao) or absent entirely (*Dictyostelium* intermediate repeat sequences; DIRS), leading to host-specific lineage expansion or total loss [1, 2].

The seven retroviral genera (alpha-, beta-, delta-, gamma- and epsilon-retroviruses, lentiviruses and spumaviruses) have variable distribution throughout Eukaryotes due to the type of exogenous retroviruses infecting the host through evolutionary time and virus tissue-specificity during infection. As a result, endogenous alpharetroviruses are only found within avian genomes [3], endogenous lentiviruses have only been found in a few mammalian genomes and there are no described examples of endogenous deltaretroviruses [4, 5]. At the time of insertion, ERVs have identical, intact LTRs capable of bidirectional transcription, and contain functional retroviral genes for *reverse transcriptase* and *integrase*, which can facilitate movement of non-autonomous repeat elements and synthesise retrogenes [6, 7]. Whilst the majority of insertions have little or no biological impact, there are many examples of LTR retrotransposons that dysregulate gene expression, become co-opted as host genes or promoters, facilitate chromosomal rearrangements and cause or modulate disease phenotypes [3, 8–11]. As genomic elements, all LTR retrotransposons are subject to the same selective pressures and evolutionary rates as the host and generally degrade over time [12].

LTR retrotransposons can comprise up to 10 % of the mammalian genome and 80–90 % of some plant genomes [13–15]. However, annotation of the current genome assembly (galGal4) of the domestic chicken (*Gallus gallus domesticus*) identifies less than 1.66 % of the chicken genome as being homologous to these elements [16]. Whilst avian repeat content is generally lower than in mammals, comparison of chicken LTR retrotransposon content with that of the turkey (*Meleagris gallopavo*) and the zebra finch (*Taniopygia guttata*) suggests that there is a deficit of these elements in Galliformes relative to Neoaves [17]. It is unknown whether this apparent deficit represents a genuine biological phenomenon or is simply due to genome assembly errors and incomplete identification.

Previous LTR retrotransposon annotation in the chicken was conservative, relying solely on sequence homology [18] or detection of putative protein-coding domains following identification by individual structural identification methods [17, 19]. More sophisticated analyses of the cow (*Bos taurus*), horse (*Equus caballus*) and dog (*Canis familiaris*) genomes has recognised both the high lineage specificity of LTR retrotransposons and the markedly different subsets of elements detected by each method [20–23]. Such an extensive classification has not yet been completed with the chicken, despite well characterised ERV variation between commercial lines and the concern of further, emergent recombinant retroviruses, exemplified by Avian Leukosis Virus (ALV)-J [24–26].

This paper describes the use of LocaTR, a newly developed LTR retrotransposon identification pipeline described herein, to create an updated annotation of the chicken galGal4 genome assembly. This pipeline is applicable to any assembled genome and utilises the best existing set of identification programs. Following initial identification, the genomic distribution of LTR retrotransposon-derived elements, their insertion age, and the extent and tissue specificity of their expression was determined. This included further characterisation of the co-opted gammaretroviral *env* gene *Ovex1*. The updated galGal4 annotation was then used to identify LTR retrotransposon content across twenty-one species in the sauropsid lineage, in a purely homology-based approach, to address the previously proposed deficit of these elements within the Galliformes.

## Methods

### Genomic resources

The galGal4 chicken genome assembly (GenBank:GCA_000002315.2) was annotated for LTR retrotransposon content using the LocaTR annotation pipeline described below. Following the analysis of galGal4, twenty-one additional sauropsid genomes were analysed for their LTR retrotransposon content using the galGal4 annotation. Of these, the nineteen avian genomes analysed were chosen for their wide phylogenetic distribution: *Anas platyrhychos* (GenBank:GCA_000355885.1), *Apaloderma vittatum* (GenBank:GCA_000703405.1) *Aptenodytes forsteri* (GenBank:GCF_000699145.1), *Calypte anna* (GenBank:GCF_000699085.1:), *Columba livia* (GenBank:GCF_000337935.1), *Corvus brachyrhynchos* (GenBank:GCA_000691975.1), *Cuculus canorus* (GenBank:GCA_000709325.1), *Falco peregrinus* (GenBank:GCF_000337955.1), *Haliaeetus leucocephalus* (GenBank:GCA_000737465.1), *Meleagris gallopavo* (GenBank:GCA_000146605.1), *Melopsittacus undulates* (GenBank:GCF_000238935.1), *Pelecanus crispus* (GenBank:GCA_000687375.1), *Picoides pubescens* (GenBank:GCF_000699005.1), *Pterocles gutturalis* (GenBank:GCA_000699245.1), *Pygoscelis adeliae* (GenBank:GCA_000699105.1), *Struthio camelus australis* (GenBank:GCF_000698965.1), *Taniopygia guttata* (GenBank:GCF_000151805.1), *Tinamus guttatus* (GenBank:GCF_000705375.1) and *Tyto alba* (GenBank:GCF_000687205.1). Two reptilian outgroups were used for comparison: *Chrysemys picta bellii* (GenBank: GCA_000241765.2) and *Anolis carolinensis* (GenBank: GCA_000090745.1).

### LocaTR — identification of LTR retrotransposons

**Fig. 1:**
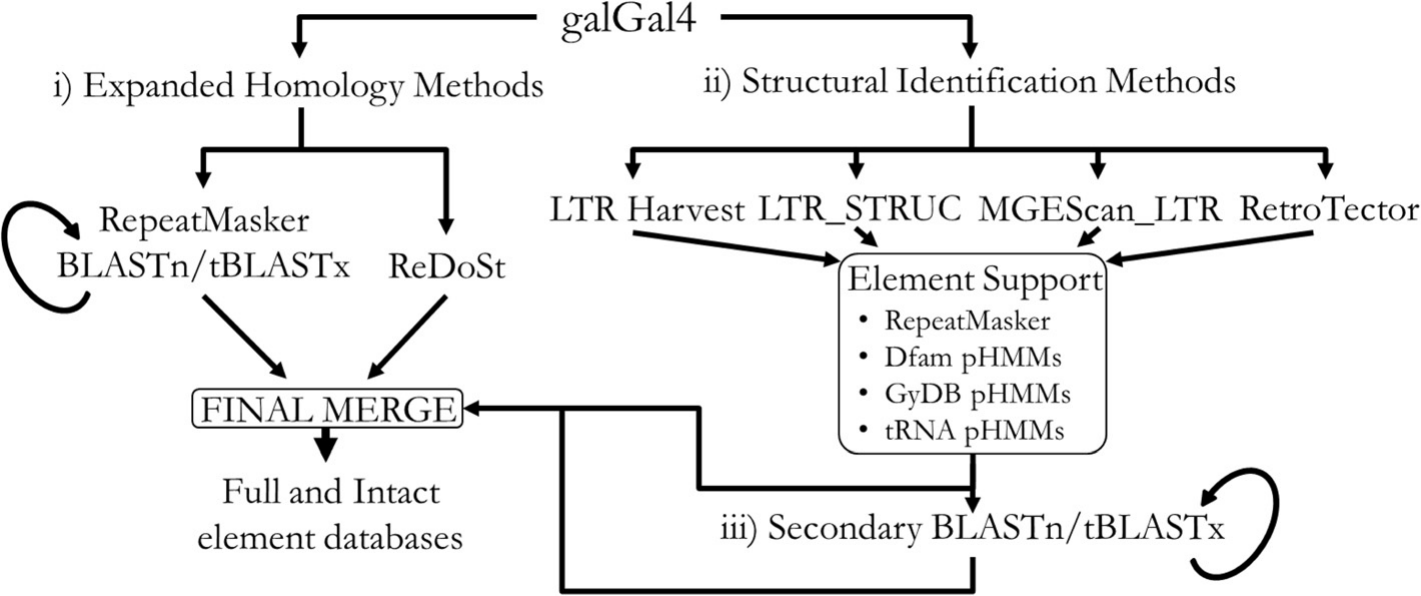
LocaTR pipeline for LTR retrotransposon identification. This flow chart shows how the structural identification, homology and secondary BLAST protocols were combined prior to the further analysis of element density, distribution, expression etc. Input/Output processing is controlled by Python and BASH scripting, and all identification programs used are freely available. The pipeline has been made applicable to any assembled genome and can be accessed via GitHub

LTR retrotransposons were identified in the galGal4 assembly using the novel identification pipeline, LocaTR. LocaTR is a user-friendly approach to LTR retrotransposon annotation, with extensive documentation and ordered, self-contained scripts, and is applicable for use on any assembled genome. The pipeline uses seven identification programs (three homology-based and four structural), linked by Python and BASH scripting for sequence pre-processing, result extraction and feature annotation. The LocaTR pipeline is shown in Fig. 1 and the three distinct identification stages outlined below.

(i) **Expanded Homology Methods:** RepeatMasker [27] analysis was performed using the “-species chicken” option, and LTR retrotransposon-annotated positions were extracted. Additionally, a library of reference LTR retrotransposons was compiled from 303 single domain and full-length sequences to be used for the extended homology-based search. This library comprised all the *Gallus* RepBase [11] entries and forty-five reference sequences from the Gypsy Database (GyDB) of Mobile Genetic Elements [28] selected for diverse LTR retrotransposon phylogenetic coverage, from avian host species where available. These custom reference database sequences were used as queries in BLASTn and tBLASTx [29] searches of galGal4, using an E-value threshold of 10^−10^, with rejection of hits shorter than 100 bp. Results were combined with the RepeatMasker output and identified retrotransposon positions were merged if they overlapped or were fewer than 11 base pairs (bp) apart. Putative annotations were analysed individually with RepeatMasker, and those with high homology to other repeat classes, such as Chicken Repeat 1 (CR1) LINE elements identified due to their *reverse transcriptase* domain, were removed. Annotations were further checked with a reciprocal tBLASTx search against the reference LTR retrotransposon library. Additionally, ReDoSt v1.1 [30] was used with default settings for the identification of the structurally divergent DIRS elements. Putative DIRS elements had to have a recognisable *reverse transcriptase* domain (E = 10^−15^ or better), and either a *methyl transferase* or *tyrosine recombinase* domain (E = 10^−12^ or better).
(ii) **Structural Identification Methods:** The conserved archetypal structure of LTR retrotransposons enables element identification independent of sequence homology, instead modelling the element based on various distance and similarity constraints. Such approaches identify LTRs by their conserved repetitive structure, as well as the presence of polyadenylation signals, transcription factor binding sites, the transcription start site, and their external demarcation by short inverted repeats. Candidate LTRs are paired based on distance and similarity constraints, and annotated pairs classified as LTR retrotransposons when additional evidence for internal motifs has been identified. LocaTR facilitates analysis with four, independently run, structural identification methods: LTR_STRUC [31], LTR Harvest [32], RetroTector [33] and MGEScan_LTR [34]. Whilst the underlying identification rationales are related, the programs have been previously found to identify markedly different subsets of LTR retrotransposons [20–23]. Differences come from the different training sequences used for modelling the LTRs, variable preferences for sensitivity and specificity during identification, and program-specific requirements for the identification of the various LTR retrotransposon domains used to confirm putative LTR pairs. Other related programs are available, but these four have a wide use in the literature and no complete identification redundancy between methods.
  LTR_STRUC v1.1 was run with sensitivity 1, per contig, to address memory issues, and then separately on reverse complemented sequence, as LTR_STRUC doesn’t consider the reverse strand during identification. Putative element positions were obtained with element BLASTn against galGal4. LTR Harvest was implemented as part of GenomeTools v1.5.1 [35] compiled with HMMER v3.1b1 [36], using default settings plus the “minlenltr 80”, “maxlenltr 2000” and “similar 75” options, based on known vertebrate LTR retrotransposon structure and testing of other user options. RetroTector v1.0.1 was used with default settings. Following the suggestion in the RetroTector documentation, contigs shorter than 30 kilobases (kb) were padded at each end to aid the identification algorithm, using the same 15 kb of randomly generated sequence with equal base frequencies. NCBI BLASTn and tBLASTx searches were performed against the non-redundant database [37] to ensure padding was devoid of gene or repeat identity. In addition to the default masking of Alu and L1 elements during the SweepDNA protocol of RetroTector, CR1 elements were also masked using an optional chicken-specific ‘broom’ developed by the RetroTector authors. Such masking was designed to limit the search space and reduce false positives. MGEScan_LTR v1.3.1 was run with default parameters.
  Following identification of putative elements by these four programs, additional support was required before they were defined as LTR retrotransposons. Each element was analysed by RepeatMasker for other repeat classes, particularly CR1. LTR retrotransposon nucleotide motifs were identified with Dfam v1.2 [38] profile Hidden Markov Models (pHMMs) using HMMER nhmmscan with an E-value threshold of 10^−5^. Protein-coding regions within the elements were identified using GyDB protein pHMMs and hmmscan with an E-value threshold of 10^−10^, following translation of each element into all six reading frames by the EMBOSS v6.6.0 transeq tool [39]. pHMMs for host tRNA genes were also built to identify the protein binding site domain. tRNA genes were identified using tRNAscan-SE [40] with default parameters, then aligned by amino acid using MUSCLE v3.8.31 [41] with default settings, and pHMMs built using HMMER hmmbuild. HMMER hmmpress was used to create pHMM flatfile databases. Results for all feature tests were assessed manually and putative elements discarded if there was insufficient evidence that they were LTR retrotransposons.
(iii) **Secondary BLAST Protocol:** All annotated structurally intact elements were used as queries for another BLASTn/tBLASTx homology protocol to identify related elements lacking archetypal structure. The final stage of the LocaTR pipeline was to combine and merge elements identified in the homology, structural and secondary BLAST protocols, resulting in the final LTR retrotransposon annotation.

### Phylogenetic and LTR retrotransposon insertion age analysis

Structurally intact elements were aligned to the forty-five GyDB reference sequences using MUSCLE with default settings, and analysed for putative coding regions with GyDB protein pHMMs, using HMMER hmmscan with an E-value threshold of 10^−10^. Domain alignments were inspected and each element was manually classified to either a retroviral genus or the Ty1/Copia or Ty3/Gypsy families of LTR retrotransposons. Protein assignments, particularly reverse transcriptase, took precedence over nucleotide alignments during classification. LTR sequence identity was calculated from MUSCLE alignment with default settings. Each element insertion was dated using nucleotide divergence rates from the Galliformes [42]. Impact of selection on element distribution over time was tested by randomly reassigning intact element insertion ages 1000 times and averaging the proportions for age categories based on LTR identity.

### Genomic distribution of LTR retrotransposons

(i) **LTR retrotransposon density and clustering:** Element density (LTR retrotransposons per Mb) was calculated per chromosome and correlated with chromosome length, gene density and average chromosomal recombination rate [43], all log_10_ transformed to normality. Pairwise Pearson correlations were performed and a General Linear Model (GLM) fitted using element density as the response variable and chromosome length and recombination rate as covariates. Data from chromosomes 27 and Z were excluded from the GLM due to large residuals in the normality plots. Furthermore, data from chromosomes 16, 25 and W could not be directly compared to the overall analysis due to substantial known sequence assembly gaps.
  Density heterogeneity was considered through identification of structurally intact elements in clusters, with a cluster defined as at least 5 elements per Megabase (Mb), compared to an even distribution of 1 element per Mb. Clusters were checked for age and genera relatedness. The probability of clusters arising by chance was assessed by comparing the observed number of intact elements within clusters to 100,000 random distributions of equal number point integrations. Differences between observed and simulated cluster numbers were quantified with exact binomial tests. Cluster recombination rate was obtained from 500 kb-average-bin data from Elferink et al. [43], following conversion of cluster positions to the Ensembl WASHCU2 (galGal3) assembly (GenBank:GCA_000002315.1) using the “Map to Reference” tool in Geneious v7.0.4 [44]. galGal4 centromere locations were also mapped using the WASHCU2 annotations.
(ii) **Distribution relative to known gene annotations:** Element locations were compared to the Ensembl galGal4 version 79 annotation file using the BedTools v2.23.0 intersectBed tool [45]. Elements overlapping with ‘transcriptional units’ (TU; regions including exons, introns, UTRs, and 5 kb up- and downstream regions, for protein and RNA genes) were annotated for strand and TU domain overlap. The shortest distance from each non-overlapping element to a TU was calculated and distances were binned in 10 kb ranges up to 100 kb. Genome-wide and per chromosome analyses were completed and compared to randomly generated simulations of equal number point integrations. Simulations were modelled using a random number generator and repeated 100,000 times. Deviation of the observed distribution from the modelled data was quantified using individual category exact binomial tests and the Kolmogorov-Smirnov test for the overall distance distributions. Similar distribution analyses were conducted relative to constrained genomic locations using two multiple sequence alignments from Ensembl: one consisting of twenty-three amniotes and another of seven sauropsids.

### RNA transcription analysis of LTR retrotransposons

Transcription of putative LTR retrotransposon was quantified using 23 diverse chicken RNAseq datasets. Of these, twenty were somatic tissues (breast muscle, bursa, cerebellum, duodenum, gizzard fat, Harderian gland, heart muscle, ileum, kidney, left optic lobe, liver, lung, ovary, pancreas, proventriculus, skin, spleen, thymus, thyroid and trachea) from the Roslin Institute chicken layer ‘J-Line’ (ENA:PRJEB12891) and three were White Leghorn chicken embryonic stages HH4-5 (GenBank:SRX893876), HH14-15 (GenBank:SRX893868) and HH25-26 (GenBank:SRX893873). All 23 datasets were quality checked with FastQC v0.11.2 [46]. All J-Line somatic tissue data were high quality, but the embryonic data exhibited low quality read ends and overrepresentation of adapter sequences. These were removed with Trim Galore v0.4.0 [47] using Cutadapt v1.4 [48].

Reads from each tissue set were mapped independently to galGal4 using Bowtie2 v2.2.5 [49] and TopHat2 v2.0.14 [50]. Inner insert size and strand orientation of all libraries was defined during mapping and then transcripts assembled using Cufflinks v2.2.1 [51] without a reference annotation. Individual RNA transcripts were overlapped with putative elements in the same orientation using intersectBed. Overlapping reads were mapped to each putative element with Bowtie2 and viewed in Geneious, where the extent of transcription for each LTR retrotransposon was assessed. Putative intact transcripts were translated into the three forward frames. Protein coding potential was assessed by both sequence homology and domain content. Intact protein candidates were used as BLASTP [52] queries against the NCBI non-redundant database and homologous results were aligned with MUSCLE and individually assessed. Putative conserved domains were identified using InterPro [53] and transmembrane topologies predicted using Phobius [54]. Patterns of selection were inferred from protein alignment using the DataMonkey [55] hosted DEPS (Directional Evolution in Protein Sequences) program [56] to predict protein regions under positive, negative or balancing selection.

### LTR retrotransposon abundance within the avian lineage

Genome assemblies of the twenty-one sauropsids detailed above were analysed for their LTR retrotransposon content using RepeatMasker, specifying the “-species vertebrates -nolow” flags. This generic analysis was extended with a second RepeatMasker analysis using a custom library built from the structurally intact galGal4 LTR retrotransposons identified here using the LocaTR pipeline. These two analyses were combined, putative elements with high homology to other repeat classes removed, and overall content presented against the sauropsid lineage [57].

## Results and discussion

### Performance of the LocaTR pipeline

**Fig. 2:**
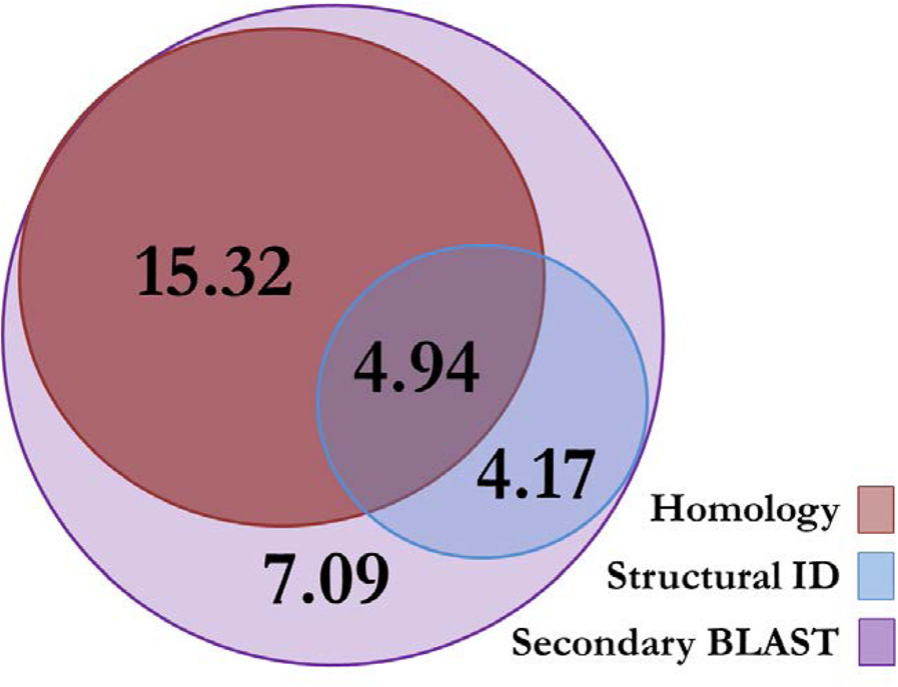
Performance of the homology and structure based identification methodologies. Euler diagram representing the relative proportion of LTR retrotransposon content identified by the homology (red), structural ID (blue) and secondary BLAST (purple) modules of the LocaTR pipeline (Fig. 1). Numbers represent total length of LTR retrotransposon sequence in megabase pairs (Mbp); 31.52Mbp in total. Homology methods identified 20.26Mbp of sequence, and structural ID methods 9.11Mbp including a 4.94Mbp (54.23 %) overlap with the homology data. The secondary BLAST annotated an additional 7.09Mbp of sequence based on elements from the structural ID search

A total of 31.5 Mb of LTR retrotransposon sequence, encompassing 3.01 % of the chicken genome, was identified using the LocaTR pipeline described here. This comprised 36,109 annotated regions of which 1,073 were structurally intact elements (SIE): more than double the number previously reported [17]. Within this set, the expanded homology protocol identified over 20.3 Mb of sequence, 4 Mb more than using RepeatMasker alone. Structural identification methods alone identified 9.1 Mb, 45.8 % of which was ‘novel’. Of the 1,073 SIEs 291 (27 %) were identified by two or more programs, but with only 7 identified by all four. With a strict two structural program identification requirement 2.8 Mb of novel annotated sequence would have been missed. LTR Harvest identified the most SIEs (643) of which 420 (65 %) were uniquely identified by this program (Table 1). Despite low cross-program corroboration, there appears to be no specific program biases for GC content, inner structural intactness, element age or genera, as had been proposed in annotation of other species [20, 21] (Table 1). The secondary BLAST analysis for fragments related to annotated SIEs identified a further 7.1 Mb of fragmented sequence (Fig. 2).

**Table 1:** Comparison of intact LTR retrotransposons identified by the four structural identification programs

	LTR_STRUC	LTR Harvest	MGEScan_LTR	RetroTector
SIEs identified	93	643	427	290
Total SIE content (bp)	767,132	4,837,212	4,928,810	2,664,622
Mean SIE length (bp)	8,249	7,523	11,543	9,188
Median SIE length (bp)	6,144	6,047	7,889	7,477
Median SIE LTR identity (%)	95.8	95.2	91.5	94.0
Mean SIE GC content (%)	48.1	47.2	45.7	46.3
SIEs unique to program (%)	28.0	65.3	44.3	50.7

SIEs identified by each program showed no significant differences in overall length, GC content, or age (shown here as mean LTR identity). All programs had SIE length distributions skewed by identification of very long LTR retrotransposons, including seven examples of nested SIEs. The high percentages of SIEs unique to each program exemplifies the necessity of using multiple identification approaches.

All three LocaTR identification stages (Expanded Homology, Structural Identification and Secondary BLAST) have an inherent bias for known sequence. Whilst this is expected in the purely homology based identification protocols, structural LTR HMMs are also biased as they are constructed from alignments of known elements. Benachenhou and colleagues [58] demonstrated that genera-specific models can be highly informative but that wider, family models, such as those used by the programs in LocaTR, have reduced specificity and increased false positive rates as a result of incorrect annotation of gene promoters as LTRs due to their conserved poly-A motifs. Given these concerns, secondary support was required for all putative elements, but again this relied upon existing nucleotide or protein motifs. As a result, it is unlikely that all chicken LTR retrotransposon-derived elements have been fully annotated here. However, it is also unlikely that those highly degraded sequences missed during annotation are of great biological relevance to the host.

### Characterisation of LTR retrotransposons

Most of the 36,109 annotated LTR retrotransposon-derived regions are fragmented, with an average length of 0.9 kb and high standard deviation (2.1 kb) reflecting the large sequence structural variability. SIEs also exhibit large element size variation (mean length 8.5 kb, standard deviation 6.5 kb), some of which can be accounted for by seven examples of ‘nested’ LTR retrotransposons, in which one element has inserted within another resulting in an elongation of the outer element. Some of the annotation improvement can be attributed to the quality of the galGal4 assembly (N50 of 279.3 kb), as previous work used galGal3 which had a four times higher proportion of ambiguous bases, a contig N50 of only 46.4 kb and significant assembly errors on the Z chromosome. However, most improvements can be directly attributed to the use of the LocaTR pipeline, due to the enriched reference sequence database and reduced conservatism during SIE identification (Table 2).

**Table 2:** Comparison of LTR retrotransposon annotations between chicken genome assemblies

	galGal3	galGal4 (RepeatMasker)	galGal4 (LocaTR)
Assembly length (bp)	1,098,770,941	1,046,932,099	1,046,932,099
Scaffold N50 (bp)	11,063,745	12,877,381	12,877,381
Contig N50 (bp)	46,345	279,750	279,750
LTR content (bp)	14,870,595	17,369,358	31,490,117
LTR content (%)	1.35	1.66	3.01
Number of SIEs	492	-	1,073

Improvements between the galGal3 and galGal4 assemblies only accounted for an extra 2.5 Mb of annotated LTR retrotransposons. LocaTR identified a further 14.1 Mb including an additional 587 SIEs not found by the RetroTector analysis of Bolisetty and colleagues [17].

A total of 65.7 % of SIEs could be classified by protein-coding domain homology. Of these, over a third were ERVs from the alpharetrovirus-betaretrovirus clade, generally the youngest elements and therefore the most easily detectable group. Consistent with previous publications, no elements were identified from either the Bel/Pao or DIRS groups of LTR retrotransposons [17, 19, 30] and there was no evidence of deltaretrovirus or lentivirus ERVs. Most of the fragments detected by the homology search were gammaretroviral in origin, likely due to the bias of mammalian sequences within the vertebrate RepBase databases.

SIEs tend to represent recent insertions and accordingly exhibit LTRs with less than 10 % sequence divergence, suggesting insertion less than 13.5 million years ago (MYA) (95 % confidence range: 12.5–14.7MYA) [57]. Nearly 90 % of all SIEs have inserted since the separation of the chicken and turkey lineages (27.0MYA, 95 % confidence range: 25.0–29.4MYA) [57]. Element GC-content (46.9 %) is not significantly higher than the genome average of 41.8 %. However, variation in element GC-content is explained by SIE insertion age, as absolute element GC-content deviance from the genomic mean decreases over time (*r* = 0.45, *p* < 0.001), as repeats are modulated by genome-specific mutation rates and substitution bias.

### LTR retrotransposon density

Element density has a strong, positive correlation with chromosome size (*r* = 0.91, *P* < 0.001), and, consequently, a strong, negative correlation with recombination rate (*r* = -0.81, *p* < 0.001) and gene density (*r* = -0.72, *P* < 0.001). Chromosome size was the only significant variable when fitted to the GLM (*P* < 0.001), but as recombination rate is scaled by sequence length (centimorgan per Mb; cM.Mb^−1^) this remains an important contextual relationship. Chromosomes 16, 25 and W exhibit much higher than expected element density, but this is likely due to large amounts of missing sequence from the assembly. However, chromosomes 27 and Z are much more complete and have high density relative to their length and recombination rate. As a sex chromosome, the Z has a very heterogeneous recombination rate with long regions of low recombination facilitating element persistence [59]. Accordingly, element density on the Z chromosome is double that of the predicted density for an autosome of equal size. Chromosome 27 element density is four times higher than on the similarly sized chromosomes 26 or 28, and includes eight SIEs. However, its overall length, GC-content and recombination rate are consistent with these neighbouring chromosomes. It also shares a 1:1 synteny with turkey chromosome 29 and zebra finch 27, so this relatively elevated LTR retrotransposon content is not the result of a recent macrochromosome fragmentation event. On close inspection, 89 % of all elements on 27 are found within 1 Mb of the 5′ telomere, a region with recombination rate fifteen times lower than the chromosomal average (0.7 cM.Mb^−1^ compared to 10.8 cM.Mb^−1^). The gene density for this region is similar to the rest of the chromosome, but the average exon number is four times smaller with many single exon ‘genes’ annotated by Ensembl as “uncharacterised, known protein coding”. Of the 40 genes in this region, 32 overlap putative elements, 28 of which with annotated exons, suggesting these ‘genes’ have been predicted from the transcription of LTR retrotransposons. As a result the “true” gene density for this region is low and more similar to observed macrochromosome levels, which, accompanied by low recombination rate, may suggest why there is a density spike in this region. Whether this is biologically accurate, or an issue with the assembly, is currently unknown and it will be interesting to analyse this region again after the release of the next chicken genome assembly.

The distribution of LTR retrotransposons on chromosome 27 is unusual compared to the overall genomic pattern, but intra-chromosomal LTR retrotransposon density is highly heterogeneous. 40.3 % of all SIEs are found within clusters (432 SIEs in 28 clusters) unrelated by insertion age or genera. This is significantly higher than expected under random integration, where only 6.49 % of SIEs would be within clusters (*P* = 1.58e^−30^). Cluster size varies from the minimum defined of 5, up to a cluster containing the 56 identified SIEs on chromosome W. Clusters are commonly associated with regions of elevated fragmented LTR retrotransposon density, suggesting the persistence of these favourable areas over time. There are also examples of regions with a high density of fragmented LTR retrotransposons linking two SIE clusters, most notably the two clusters on chromosome 2. All clusters are in regions of low recombination relative to the chromosomal average (including clusters on chromosomes 1, 2, 4, 5 and 8 which encompass the centromere), which likely aids the structural longevity of elements in these regions. As a converse example of the relationship between density and recombination rate, the highly recombining pseudoautosomal region of the Z chromosome has just 2.7 % of the expected LTR retrotransposon sequence given its length and the chromosomal element density.

### LTR retrotransposon distribution

**Fig. 3:**
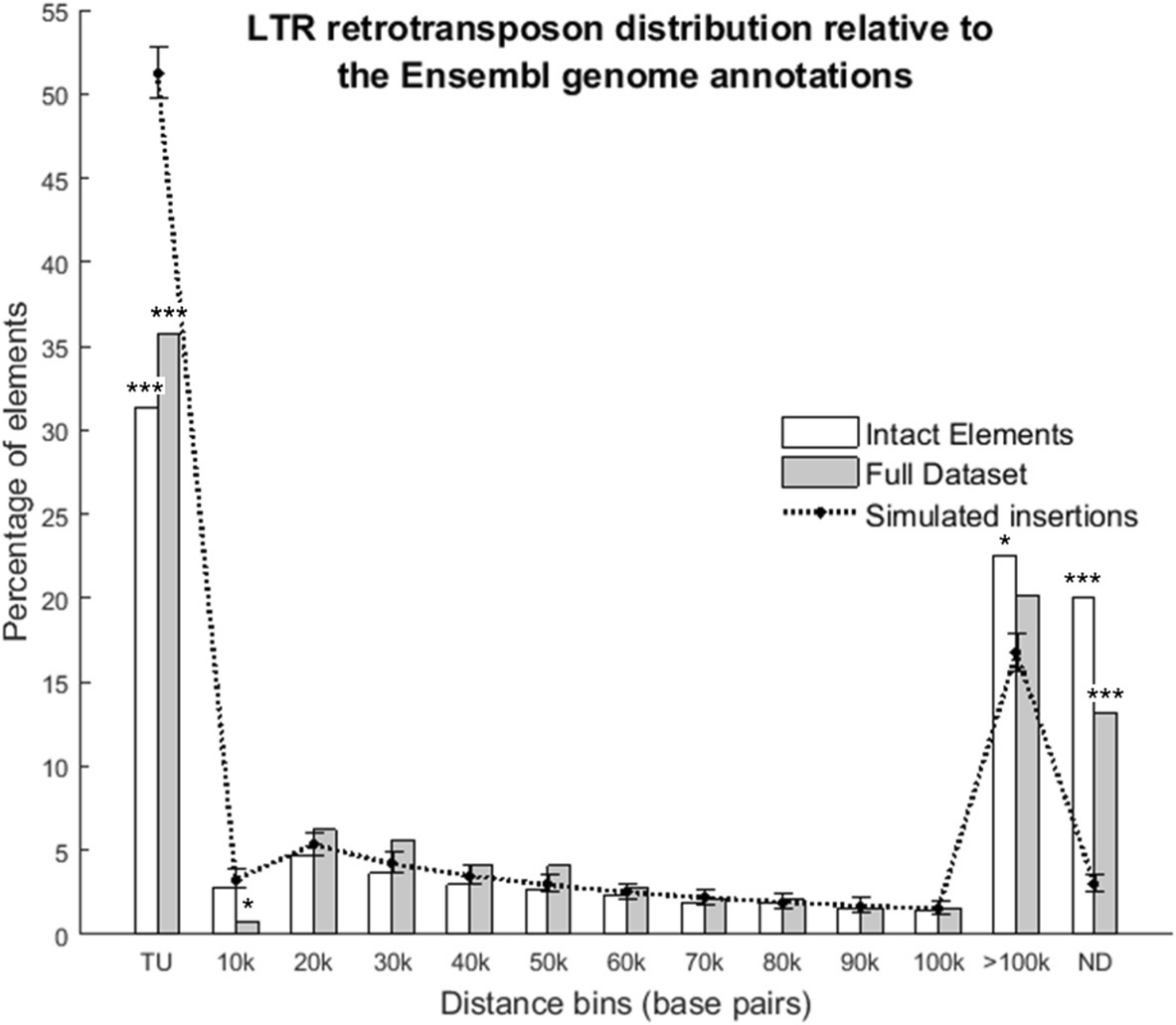
LTR retrotransposon distribution relative to Ensembl genome annotations. Distances of LTR retrotransposon for both the full (red) and structurally complete (blue) data sets, from Ensembl annotations. The genome wide distribution has significant depletion of elements in the TU for both full (*P* = 1.94e^−5^) and structurally complete (*P* = 3.90e^−4^) lists. Significance is highlighted using asterisks. Distances are the shortest intergenic distance between element and Ensembl annotation measured in 10 kilobase bins (where the value is the bin upper limit). TU = Transcriptional Unit (incl. exons, introns, UTRs and flanking 5 kilobases up and downstream). ND = Non-Defined (elements on contigs without any Ensembl annotation). Plot was constructed with MATLAB R2015b

Assuming random integration, 51.3 % of all LTR retrotransposons would be expected within TUs, of which 87.3 % would be within introns. However, there is a significant depletion of LTR retrotransposons in TUs in both the full data set (31.3 %; *P* = 1.94e^−5^) and SIE data sets (35.8 %; *P* = 3.90e^−4^) summarised in Fig. 3. This skewed result is observed with the overall distance distribution with both full (KS = 0.139, *P* = 1e^−100^) and SIE (KS = 0.146, *P* = 1.28e^−17^) datasets exhibiting significant shifts away from the TU. Taken genome-wide, some of the detail is overlooked: microchromosomes generally follow the random integration distribution, but the longer macrochromosomes exhibit the depletion of elements within the TU (e.g. chromosome 1; 28.3 % compared with 47.2 % under random distribution; *P* = 3.88e^−5^). Additionally, chromosomes 1–5, 8 and Z have significant enrichment of elements greater than 100 kb away from TUs (e.g. chromosome 1; 42.8 % compared with 23.0 % under random distribution; *P* = 1.17e^−5^). These chromosomes are also gene sparse and contain 73.6 % of the clustered SIEs between them (318 elements in 20 clusters); elements within clusters are significantly depleted within TUs (*P* = 1.6e^−7^) and enriched greater than 100 kb from TUs (*P* = 0.01) relative to the observed SIE distribution.

Together these data suggest that new insertions within TUs are selected against, and that accumulation is tolerated primarily in the poorly recombining, gene sparse regions of the genome where clusters can form and persist over long evolutionary timescales due to low selective constraints. Consequently, SIE distribution should be age dependent, with new insertions following a random distribution and older elements skewed away from TUs. Whilst there is some evidence that SIEs within clusters are generally older than those outside, there is no suggestion that SIE age distribution differs from randomly generated redistributions. Analysis is, however, confounded by the dominating proportion of “young” SIE insertions (70 % LTR identity or greater; 96.52 % of all SIEs). Older elements alone exhibit depletion in TUs and enrichment greater than 100 kb away from TUs, but the sample size is too small for statistical robustness.

Despite selection, 31.3 % of all elements and 35.8 % of SIEs overlap TUs. However, as described above with chromosome 27, it is clear that some of these overlaps are due to errors in the Ensembl gene predictions. Random distribution would suggest that only 4.9 % of those elements overlapping TUs should be in exons, but the full data set has significant enrichment of exon overlaps (10.1 %; *P* = 0.015). This effect is greater with the SIEs: enrichment in exons (38.3 %; *P* = 4.1e^−24^) and the 5′UTR (5.5 %; *P* = 0.005), and subsequent depletion of intron overlap (51.6 %; *P* = 1.3e^−18^). There is no significant sense/antisense difference as had been previously reported [17].

It is, however, unlikely that all exon overlaps are a result of incorrect annotation. The two sets of constrained positions provide some evidence of biological significance: 238 SIEs contain at least one constrained element and of these 82 have constrained elements from both lists, 82.9 % of which overlap exons. Such overlaps may be representative of retrotransposon-derived exons or regulatory regions, or potential false positives during identification. All studied cases lack any LTR retrotransposon homology through BLAST or pHMM analysis, even if surrounding regions may have some fragmentary homology. Overall, 29 LTRs (representing 21 different SIEs) contain constrained elements, but again these elements largely overlap annotated exons, rather than being standalone promoters under selection. Most constrained element overlap appears to be related to internal element regions relating to expression. Notably, none of the eight SIEs on chromosome 27 contain constrained elements. In addition, there is significant under-representation of both the sauropsid (*χ*^2^ = 8.41; *P* = 0.004) and amniote (*χ*^2^ = 3.95; *P* = 0.047) constrained elements within clusters.

### Evaluation of SIE RNA transcription data

A total of 379 (35.3 %) SIEs have detectable RNA expression in the correct orientation, within robust transcript models, in at least one of the twenty-three tissues analysed. Expression is not biased towards younger elements or specific genera, but only 24.8 % of expressed SIEs are found within clusters. Expressed elements appear to follow a random distribution pattern relative to the Ensembl annotation, but those that overlap TUs are highly enriched in exons (47.1 %; *P* = 4.1e^−24^). Only 31 SIEs exhibit “complete” expression, defined as a transcript extending at least the element length without the LTRs. Again there is no apparent bias for genera and insertion age.

Two thirds of all complete transcripts can be found in at least one embryo stage, but there is no evidence for significantly elevated expression at the earliest stage. Incomplete transcription of LTR retrotransposons across all 379 SIEs suggests a gradual decline through these three embryonic stages studied (186, 168 and 144 SIEs expressed respectively). Pancreas and ovary are the most represented somatic tissues (each had 143 elements with at least fragmented expression, with 49.7 % overlap between the two tissues), but have the 6^th^ and 15^th^ highest transcript model coverage of the genome. This suggests element expression may be tissue specific, rather than simply related to how much RNA a specific tissue expresses across the genome.

Most identified LTR retrotransposon transcripts have high frequencies of closely interspersed stop codons in all three translated forward frames. However, there are examples of potentially full length protein products. One SIE on chromosome 8 (chr8:10499515–10505342) has a long open reading frame (ORF) with high homology to betaretrovirus *pol* polyprotein and is expressed in embryo stages 14–15 and 25–26. A second SIE (chr4:85449603–85458772) has two long ORFs in the 1^st^ frame with high homology for *gag* and *pol* polyproteins respectively, and a third long ORF in the 2^nd^ frame with high homology for the *env* polyprotein, all from gammaretroviruses. Whilst the *gag* and *pol* putative proteins appear truncated and lack some domains (although reverse transcriptase appears intact in all cases). The *env* ORF encodes Ovex1 (GenBank:NP_001159385.1), a previously described protein of known retroviral origin [60].

BLASTP searches identified homologous Ovex1 proteins in many other bird species including turkey (GenBank:XP_010708895.1, 1e^−200^, 95 % identity), duck (GenBank:XP_012958629.1, 1e^−200^, 86 % identity), 18 Neoaves and even four reptiles (*Anolis carolinensis*, *Python bivittatus*, *Thamnophis sirtalis* and *Pelodiscus sinensis*). The avian sequences are generally well conserved at the 3′-end and three species (*Anser cygnoides*, *Serinus canaria* and *Zonotrichia albicollis*) have slightly divergent duplicate proteins, again with greatest conservation at the 3′-end.

In their characterisation, Carré-Eusèbe and colleagues [60] determined that chicken *Ovex1* RNA expression was limited to the gonads, but the RNAseq analysis described herein supports ubiquitous expression, with full-length transcript models generated for ten adult tissues, including the ovary, and stage HH4-5 in the embryo data. Furthermore, the other ten adult tissues studied have expression across the region, but at a level below the threshold required for transcript model construction in Cufflinks. Whilst expression in the ovary is highest in this analysis (over 1000 times more read support than in spleen; the least supported, intact transcript model), *Ovex1* expression is clearly not limited to gonad development and may have a more general function.

InterPro analysis identified one transmembrane (TM) domain near the protein carboxyl-terminus (corroborated by the Phobius analysis which also suggested the first 825 amino acids are non-cytoplasmic) and several putative protein-protein interaction domains. 207 sites of the avian Ovex1 homologue protein alignment (18.8 %) exhibit purifying selection, including sites throughout the TM domain, supporting the observed protein 3′-end conservation throughout the avian lineage. Similar analysis of exogenous retroviral *env* sequences closely match these features, but with an extra, more 5′-TM domain. In exogenous retroviruses transcribed *env* is spliced into its constituent ‘surface’ and ‘transmembrane’ domains, which then form a heterodimer after translation, and a subsequent homotrimer of heterodimers to form the retroviral envelope. Other examples of host co-opted gammaretroviruses, notably the mammalian *syncytin* placental genes and murine antiviral receptor interference genes *Fv4* and *Rcmf*, form this homotrimer for their host function [61, 62]. The presence of putative protein-protein interaction sites on the Ovex1 protein suggests that it could also form these functional homotrimer complexes. Cell-cell cohesion, similar to that effected by the *syncytin* protein, seems unlikely due to the ubiquity of tissue expression. It is more likely that the protein may have a role in innate antiviral immunity through receptor interference, as has been widely documented in mouse (*Mus musculus*) and cat (*Felis catus*) with gammaretroviral *env*, in sheep (*Ovis aries*) with betaretroviral *env* and in chicken with alpharetroviral *env* [62–66], by physically blocking retrovirus entry sites as a competitive inhibitor. Identification of duplicated homologues in three avian species also supports receptor interference, with duplicates selected for defence to related, but different exogenous gammaretroviruses. This would be the first example of gammaretroviral *env*-mediated receptor interference in chicken.

No putative proteins were identified from alpharetroviral elements. However, some of the many, largely intact alpharetroviral LTRs could provide the basis for novel or alternative promoter activity, as could those of other retroviral groups. All intact *pol* polyproteins from the two expressed SIEs described above have recognisable reverse transcriptase and RNaseH domains, which suggests they have retained the ability to transpose other repeats, including non-autonomous elements. Incorporation of reverse transcribed genomic mRNA can form retrogenes; elements with huge evolutionary potential for the host through the introduction of intact domains to existing genes, or full gene duplication [7]. The expression of intact, likely functional, retroviral domains also presents more opportunity for recombination with exogenous retroviruses. Identification here of both gamma- and betaretroviral expressed domains, rather than just the alpharetroviral elements implicit in the formation of ALV-J [24, 25], also extends the range of potential recombinant viruses, especially as cross-genera recombination has already been observed [26].

### LTR retrotransposons in the avian phylogeny

**Fig. 4:**
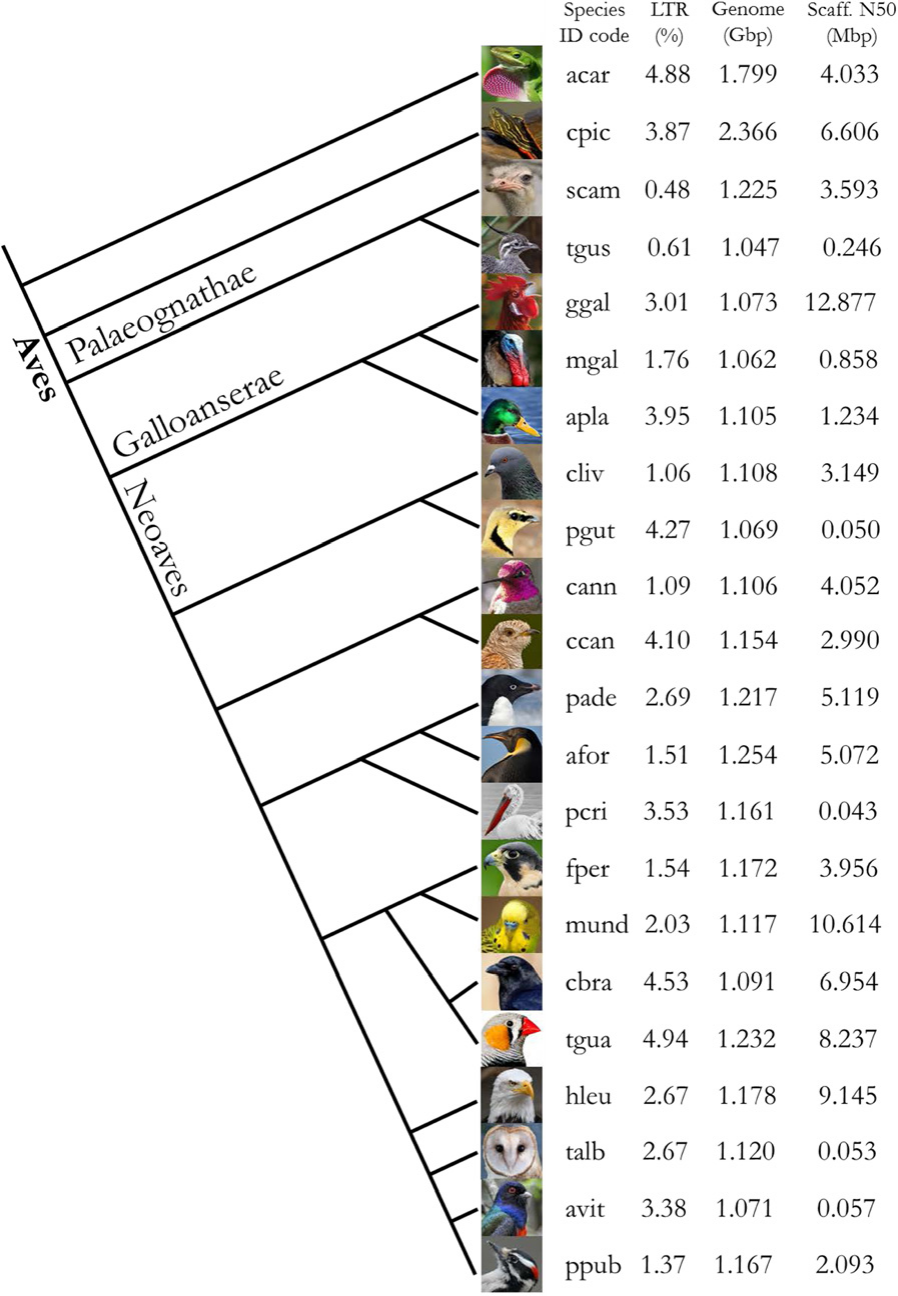
LTR retrotransposon genome content across the Avian Lineage. Avian lineage cladogram showing twenty species from the three major lineages of birds and two outgroup species: the Carolina anole and the Western painted turtle. The LTR (%) column shows the relative proportion of the genome annotated as LTR retrotransposon by the combined RepeatMasker protocol. The third column gives the genome size in gigabase pairs (Gbp). The final column gives the scaffold N50 in megabase pairs (Mbp), as a measure indicative of assembly quality. Cladogram constructed based on the avian phylogeny constructed by [57]. Species names are reported as four letter codes in column 1. From top to bottom these are: acar (*Anolis carolinensis*), cpic (*Chrysemys picta bellii*), scam (*Struthio camelus australis*), tgus (*Tinamus guttatus*), ggal (Gallus gallus), mgal (*Meleagris gallopavo*), apla (*Anas platyrhychos*), cliv (*Columba livia*), pgut (*Pterocles gutturalis*), cann (*Calypte anna*), ccan (*Cuculus canorus*), pade (*Pygoscelis adeliae*), afor (*Aptenodytes forsteri*), pcri (*Pelecanus crispus*), fper (*Falco peregrinus*), mund (*Melopsittacus undulates*), cbra (*Corvus brachyrhynchos*), tgua (*Taniopygia guttata*), hleu (*Haliaeetus leucocephalus*), talb (*Tyto alba*), avit (*Apaloderma vittatum*), ppub (*Picoides pubescens*)

For all species, use of the updated galGal4 annotation described above increased the amount of annotated LTR retrotransposon content relative to the RepeatMasker vertebrate library analysis alone. However, species more genetically distant from chicken gained less additional sequence from inclusion of the galGal4 annotation. LTR retrotransposon content is heterogeneous (Fig. 4) and does not correlate with either phylogenetic position, genome size or scaffold N50 size. Furthermore, evolutionarily close species can exhibit large differences in LTR retrotransposon content, such as hummingbird (cann) and cuckoo (ccan) (Fig. 4). Altogether this suggests extensive lineage specificity due to novel insertions and expansions or constriction of LTR retrotransposon families. This hypothesis is supported by the zebra finch exhibiting the highest LTR retrotransposon content within the Neoaves [17]. Based on this dataset, there is no clear difference in LTR retrotransposon content between the Galloanserae and Neoaves. Furthermore, when accounting for the differences in avian and mammalian genome sizes, there is no difference in relative LTR retrotransposon content. Future work should expand this lineage analysis to all sequenced birds and more reptilian outgroups, and use the full LocaTR pipeline to remove any homology approach bias.

## Conclusions

This updated analysis of the LTR retrotransposon content of the chicken genome has nearly doubled the previously reported genomic levels and now includes 1,073 structurally intact elements. This is mainly attributed to the use of LocaTR, a newly developed pipeline, which uses seven identification programs (three homology-based and four structural) to provide an extensive annotation of LTR retrotransposon content. LocaTR facilitates identification of more divergent or degraded sequences whilst accounting for the inherent biases of different individual approaches. LocaTR provides a user-friendly LTR retrotransposon identification process, with clear documentation and ordered intermediary scripts clarifying the nuances of the individual identification programs, without obscuring their own extensive customisation. LocaTR can be used to analyse any assembled genome and can be adapted to include additional identification programs, if required. The updated chicken annotation has also facilitated an extended classification of LTR retrotransposons across the avian lineage beyond existing avian comparative genomic analyses [67], and suggests that there is no real deficit of these elements in the Galliformes. Additionally, avian genomes have similar LTR retrotransposon content to mammalian genomes when scaled by the three-fold difference in genome size. Future work will more widely characterise the avian lineage with full LocaTR analysis.

Detailed analysis of the chicken LTR retrotransposons has shown that element distribution is non-random, with significant depletion of elements within coding regions and enrichment of element density in gene sparse areas. Over 40 % of elements are within clusters unrelated by age or insertion date. Bolisetty and colleagues [17] proposed that LTR retrotransposon clusters in chicken had roles as cytoskeletal binding regions or as hotspots for recombination. This new analysis has, however, found no evidence for constraint within cluster locations and that most clusters have low or negligible recombination rates. Genomic distribution of elements is, therefore, dependent on insertion neutrality, as non-detrimental elements are retained producing skewed distributions away from coding regions. Clusters form as insertion areas with low host impact increase, eventually self-perpetuating over time. This concept may have wider impact on genome size, as high repeat content enables repeat content expansion, a concept already explored in the comparison of avian and mammalian genome stability [68, 69], and may explain why avian genomes initially appeared to have a deficit of LTR retrotransposons relative to mammals unless scaled by genome size.

Transcribed LTR retrotransposons in the chicken are rare, and even these examples are largely fragmented or code for non-functional proteins. However, the identification of intact *gag*, *pol* and *env* transcripts, with apparent tissue specificity, is of great interest. The analysis herein has extended the understanding of the potential role of the gammaretrovirus derived *Ovex1* gene, due to its much more ubiquitous expression pattern than had been previously described. Whilst the biological function of this protein is currently unknown, it is possible that it forms a functional homotrimer with antiviral functionality through receptor interference.

Transcribed ORFs, a widely expressed co-opted gene and the high abundance of intact LTRs exemplifies the impact LTR retrotransposons can have within the chicken genome. However, whilst this updated annotation is a snapshot of LTR retrotransposon abundance in the chicken reference genome, extensive diversity of these elements is well documented between commercial chicken lines [25, 70]. Future work will use this annotation to quantify the extent of LTR retrotransposon diversity in multiple commercial lines and assess the impact of any novel insertions or structural variants identified.

## Additional file

Additional file 1: galGal4 LTR retrotransposon locations. Positions are in BED6 (Browser Extensible Data 6-column) format with column definitions: chromosome name, element start coordinate (zero indexed), element end coordinate, element description (FL = “full list”, SIE = “structurally intact element”), score (arbitrary and always set to ‘0’), element strand (+/-). (BED 1002 kb)

## Abbreviations

CR1: Chicken repeat 1
DIRS: *Dictyostelium* intermediate repeat sequences
Env: Envelope gene
ERV: Endogenous retrovirus
GLM: General linear model
kb: kilobase
kbp: kilobase-pair
LTR: Long terminal repeat
Mb: Megabase
ORF: Open reading frame
pHMMs: Profile hidden markov models
SIE: Structurally intact element
TM: Transmembrane
TU: Transcriptional unit
